# Prolonged Use of Surgical Masks and Respirators Affects the Protection and Comfort for Healthcare Workers

**DOI:** 10.3390/ma15227918

**Published:** 2022-11-09

**Authors:** Xiaoning Guan, Jing Lin, Jiaxiang Han, Xiaodong Gao, Ying Zhang, Bijie Hu, Robert Guidoin, Lu Wang

**Affiliations:** 1 Key Laboratory of Textile Science and Technology, Ministry of Education, College of Textiles, Donghua University, Shanghai 201620, China; 2 Key Laboratory of Textile Industry for Biomedical Textile Materials and Technology, Donghua University, Shanghai 201620, China; 3 Department of Hospital Infection Management, Zhongshan Hospital, Fudan University, Shanghai 200032, China; 4 Department of Infectious Diseases, Zhongshan Hospital, Fudan University, Shanghai 200032, China; 5 LOEX, Division of Regenerative Medicine, Centre de Recherche du CHU, Université Laval, Quebec City, QU G1V 0A6, Canada; 6 Department of Surgery, Faculty of Medicine, Université Laval, Quebec City, QU G1V 0A6, Canada

**Keywords:** surgical mask, respirator, long-term wear, safety, comfort

## Abstract

This study explored the ideal period for wearing masks to prevent the physiological and psychological problems associated with long-term face mask use during respiratory infections by healthcare workers. Breathing simulators, surgical masks (SM) and medical respirators (PM) were prepared for two to eight hours. Changes in the comfort of masks (facial skin temperature, breathing resistance, and moisture permeability) and protection (filtration efficiency, resistance to blood penetration, and colony count) were assessed. The results demonstrated that the masks offered efficient liquid-particle filtering even after eight hours of use. However, the number of bacterial colonies using PM and SM grew significantly after two and four hours, respectively. Concerning comfort, the inspiratory resistance of masks rose dramatically after two hours, whereas the moisture permeability declined considerably after four hours. In addition, skin temperature had a significant increase within two hours, which may result in facial discomfort. When conditions permitted, the hospital staff was instructed to replace their masks every two hours.

## 1. Introduction

This century witnessed the emergence of four fatal infectious respiratory diseases: SARS, swine flu, MERS, and coronavirus disease 2019 (COVID-19). Since the year 2020, COVID-19 has rapidly spread around the world [1], posing a global health threat [2]. As the virus traveled, it mutated and evolved into versions that could evade the immune response in the human body and threaten to strain health systems in the world [3,4]. The primary mechanisms of COVID-19 transmission [5,6] are direct or indirect contact, aerosols, and droplets caused by speaking and coughing. The correct use of personal protective equipment (PPE), including masks, is necessary to limit nosocomial spreading [7] and combat COVID-19 transmission [8,9].

With the onset of the COVID-19 pandemic, the selection process to wear procedure masks on various occasions has become more evident, and numerous experts have presented suitable recommendations for their use [10,11,12,13]. In addition, there is related documentation about the duration of using them. According to a publication by the General Office of the National Health Commission [14], it is customarily required to wear a medical and protective face mask for four hours when undertaking activities that may generate aerosols. Urgent replacement is needed in the event of contamination and moisture. In April 2020, the World Health Organization suggested that if there is a shortage of medical/surgical masks, they could be worn for six hours when caring for patients with COVID-19. Subsequently, the World Health Organization and the International Labour Organization noted that in the context of COVID-19, intense workloads, patient flows, and PPE shortages could necessitate healthcare workers to wear masks for extended periods [15].

However, based on interviews and studies, medical experts are unsure about the exact period of mask use. According to studies, masks (Type IIR medical face masks) could be worn for 8 h without a substantial reduction in bacterial filtration, breathability function [16] and particle filtration efficiency [17]. In a survey of nurses who wore masks (N95 respirators), 90% (n = 9) were able to tolerate them for two 12 h shifts. Although many subjective complaints were recorded, their long-term usage did not cause any clinically relevant physiological burden [18]. In contrast to the findings of this study, it has been demonstrated that prolonged use of masks (KN95 respirators, gauze masks, medical surgical masks, etc.) had a substantial impact on the emotional and physiological responses of individuals it greatly aggravated the discomfort [19]. Long-term usage of the masks (N95 respirators) by healthcare workers may result in alterations in gas exchange [20]. Short-term use of the masks (N95 respirators) during high-intensity physical activity may affect physiological changes in CO_2_ and O_2_ [21]. The accumulation of heat and moisture creates a microclimate inside the mask (N95 respirator) [22]. Itching, rashes, redness, and pressure bruises might result from long-term mask use [23,24,25]. A nasal bridge scarring and facial itching occurred in one study where masks (N95 respirator) were worn nonstop for over 12 h [26]. The potential risk typically connected with cutaneous side effects was prolonged PPE use [27]. Even headaches had been linked to wearing masks (N95 respirators) for more than four hours per day [28]. Until recently, there has been a lack of studies comparing the effectiveness and comfort brought by masks when worn for varying amounts of time (e.g., from zero to eight hours). Due to the lack of comprehensive studies, there is still uncertainty surrounding mask-wearing recommendations. Does the prolonged use of masks by health workers affect their protective efficacy? What is the balance between protection and comfort?

Therefore, the purpose of this study was to determine the optimum time for healthcare workers to wear different masks, by means of assessing the overall changes in protection and comfort of the masks after various wear times. Following the study, two types of masks—surgical masks (SM) and medical respirators (PM)— that were frequently used by medical staff were chosen. A breathing simulator was utilised by simulating the frequency, temperature, and humidity of human breathing. Starting with the safety and comfort of the masks, this study evaluated the relationship between safety performance (filtration efficiency, resistance to blood penetration, and the total number of bacterial colonies) and comfort performance (face temperature, respiratory system resistance, and moisture permeability) over time of wear. The finding demonstrated the optimal time to wear a mask, which would help healthcare personnel use them properly, reduce their psychological stress, and lessen the health hazards associated with inappropriate wear.

## 2. Materials and Methods

### 2.1. Experimental Sample

**Figure 1:**
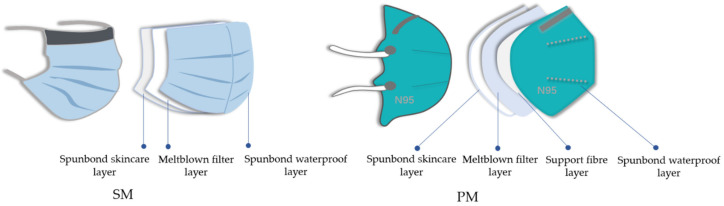
Schematic diagram representing the whole multilayer structure of the masks.

Surgical masks (SM) and medical respirators (PM) worn by medical staff nowadays were chosen as research subjects. Table 1 provides the basic characteristics of two types of masks. Figure 1 depicts a schematic diagram of their multilayer structure. A surgical mask (SM) consists of three layers, whereas a medical respirator (PM) has four.

### 2.2. Test Method

**Figure 2:**
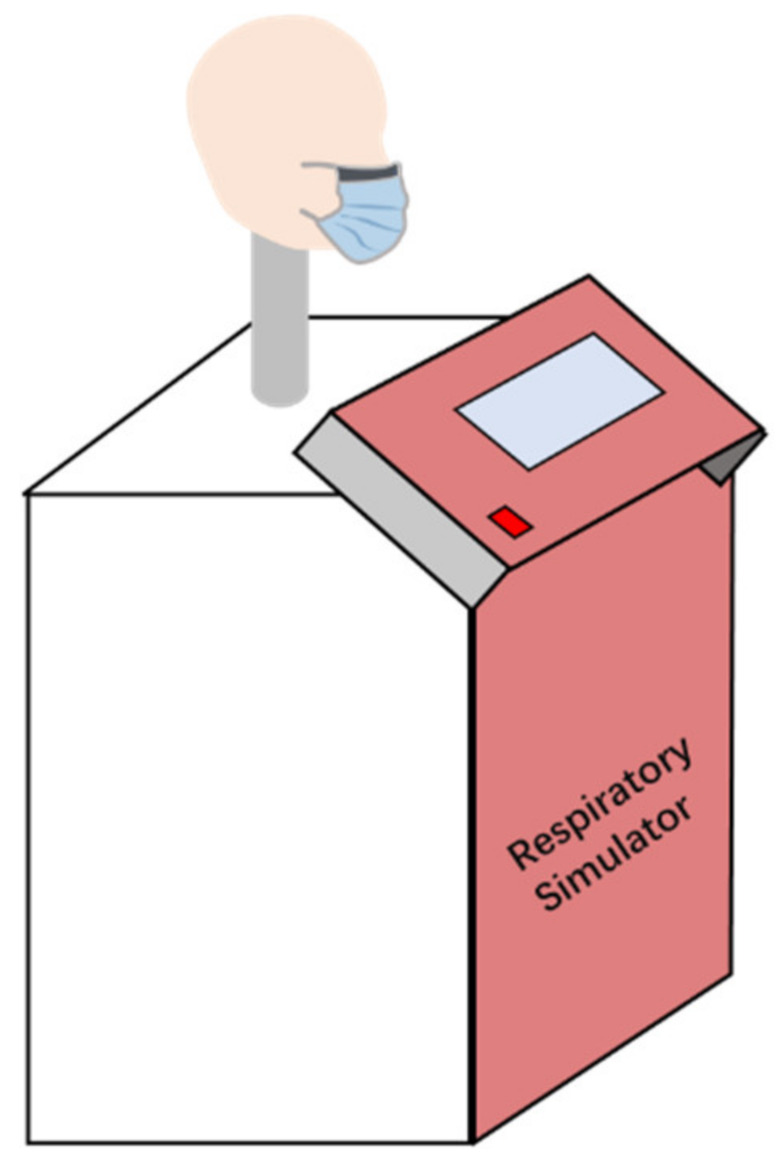
Simulation of wearing masks for different durations.

Figure 2 illustrates the simulated wearing of a surgical mask (SM) and a medical respirator (PM) for 2, 4, 6 and 8 h, respectively, using the breathing simulator. The respiration simulator was performed at 20 breaths/min with an airflow rate of 2 L. The temperature of the saturated water vapour near the mouth of the head model was 37.0 ± 2.0 °C. The simulation was conducted in a laboratory where the temperature and humidity were kept constant at 20.0 ± 2.0 °C and 65.0 ± 4.0%, respectively.

#### 2.2.1. Protective Properties

- Filtration efficiency

According to the standard GB 19083, the filtration efficiency of non-oily particles in masks was tested after varying simulated wearing times. With a flow rate of 85 L/min, the Automated Filter Tester 8130 (TSI Corporation, Shoreview, MN, USA) was utilised. The particle size of sodium chloride was 0.3 μm. A specialized mask mold was used to ensure that sodium chloride particles pass only through the mask’s surface without escaping through the edges. After three repetitions of each test, the average result was determined.

- Synthetic blood resistance of mask surface

The masks that underwent different wearing times were evaluated for anti-synthetic blood penetration in compliance with YY/T 0691 and YY 0469. The JYF-247X Medical Face Mask Synthetic Blood Penetration Tester (Shanghai JIFA Instrument Equipment Co., Ltd., Shanghai, China) was used to simulate 16 kPa of blood pressure in humans. The injection speed was set at 550 cm/s. When injecting, a volume was sprayed at the central part of the mask and after 10 min, its back was checked for signs of penetration.

- Total bacterial colony count

Different individuals wore SM and PM in a laboratory at constant temperature and with relative humidity for various periods. In accordance with GB 15979, they were tested for bacterial contamination. Samples were taken from nose and mouth masks and diluted 1:20 with saline. After thorough mixing, the aforementioned salt samples precipitated spontaneously. Supernatants were collected for colony counting; 1 mL of sample solution was applied to each Petri dish, followed by 15–20 mL of nutrient agar medium. After mixing and hardening, plates were inverted and incubated for 48 h at 37 °C. 

#### 2.2.2. Comfort Properties

- Infrared imaging of facial skin temperature

An Infrared Thermal Imager T630sc (FLIR, Wilsonville, OR, USA) was utilised to record thermal images of a head model wearing a SM or a PM to simulate normal breathing for a predetermined time duration. The FLIR ReserchIR Max software (version 4.40.9.30) could calculate the average temperature of a specific spot or region on the face in response to fluctuating skin temperature during different wearing periods.

- Respiratory system resistance

As required under GB2626, the breathing resistance of masks was evaluated after simulated wearing for different periods. As the automatic equipment for breathing resistance tests, the JYF-246T (Shanghai JIFA Instrument Equipment Co., Ltd.) was chosen and its gas flow rate was set at 85 L/min. During testing, the gaps between the face and the mask were sealed with adhesive tape to prevent any edge leakage. Following five repetitions of each test, the mean result was determined.

- Moisture permeability

The moisture permeability of masks that underwent different wearing duration was measured in accordance with the standard GB/T 12704. A temperature of 38 °C, relative humidity of 90%, and gas flow rate at 0.5 m/s were applied to the Water Vapour Permeability Tester FX 3180 (Textest, Schwerzenbach, Switzerland). Random samples were taken from the same region of multiple masks to avoid contacting the desiccant. The average value was taken as the result following the three repetitions of each test.

#### 2.2.3. Statistical Test

Using IBM SPSS Statistics 25, statistical analysis was carried out. Hypotheses were tested using ANOVA and multiple comparisons. A significant difference was indicated by *p* < 0.05. 

## 3. Results and Discussion

### 3.1. Filtration Efficiency

**Figure 3:**
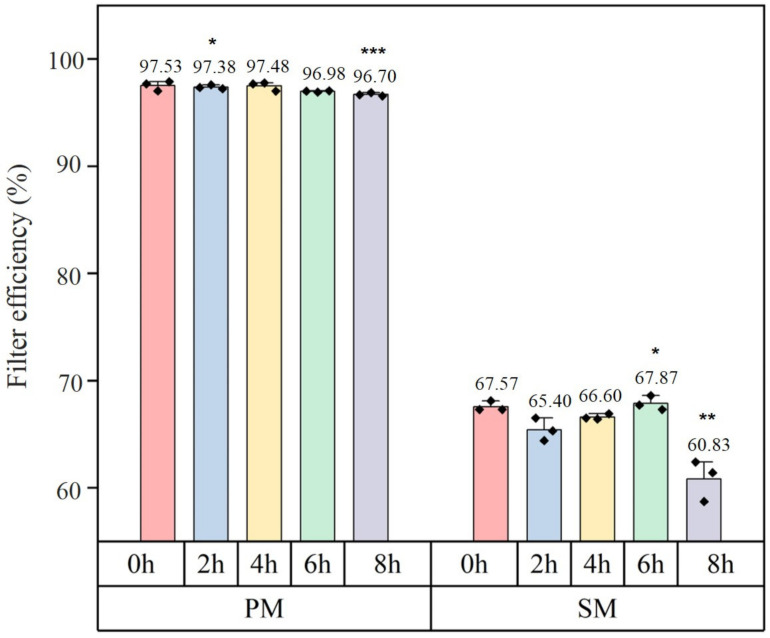
Results of filtration efficiency on particulate matter. ◆ represents a data point. *: *p* value < 0.05, **: *p* value < 0.01, ***: *p* value < 0.001.

Filtration effectiveness varied with wear time for the two types of masks, as seen in Figure 3. From zero to eight hours, the filtration efficiency of surgical masks (SM) and medical respirators (PM) declined gradually, reaching 1.04% and 9.97%, respectively. After eight hours, PM effective at filtering might still exceed 95%, effectively blocking 95% of particulate matter and providing excellent protection. After eight-hour use, the filtration efficiency of the two masks drastically decreased. 

**Figure 4:**
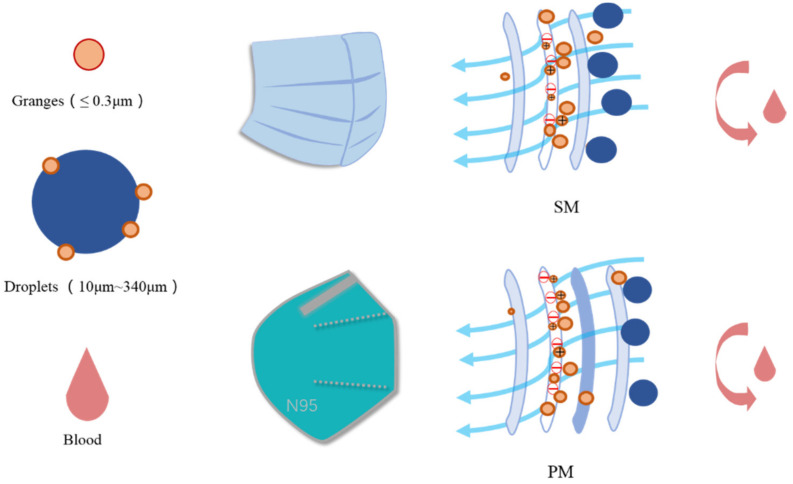
Diagram of mask filtration.

Spunbond + Meltblown + Spunbond (SMS) constructions for surgical masks consisted of a spunbond nonwoven outer layer, a meltblown nonwoven middle layer and a spunbond nonwoven inner layer [29]. To form the SMMS structure, the respirator added a filter support layer to the SMS. A mixture of five mechanisms affected the efficacy of the mask’s filtration [30]: gravitational settling, direct interception, inertial interception, diffusive interception and electrostatic interception. According to related investigations [31,32,33,34], the COVID-19 virus had a diameter from 0.06 to 0.14 μm, whereas the particle size of droplets produced from speaking, coughing, and sneezing ranged from 10 to 340 μm. The most penetrating particle size in masks was 0.3 μm [33]. Electrostatic forces would capture viruses or contaminants smaller than 0.3 μm as they pass through the pores between the fibre webs. They were unable to continue moving ahead, as depicted in Figure 4. Electrostatic adsorption played a crucial role in filtering out germs and viruses [34]. Most meltblown layers utilised electret technology [35,36] to generate electrostatic charges. This makes the layers better at filtering without making the air resistance worse. Increasing humidity accelerates charge degradation [37]. When relative humidity exceeds 55%, the rate of charge decay for the PP material utilised in the majority of masks accelerates dramatically. Constant breathing caused the mask to be filled with moist, warm air, causing the relative humidity to increase. The increased humidity caused the filter layer to lose a significant amount of its electrostatic charge, hence diminishing its ability to capture electrostatic charges and decreasing its filtering efficiency. Wearing the mask for longer than six hours is not advisable due to reduced filter efficiency.

### 3.2. Synthetic Blood Resistance of Mask Surface

The preparation of synthetic blood conformed to the YY 0469 standards. It contained sodium carboxymethyl cellulose, Tween-20, sodium chloride, methylisothiazolinone, red dye amaranth, and distilled water. Its surface tension and viscosity were comparable to those of blood, as its hue resembled it. Table 2 shows the findings on testing the mask resistance to synthetic blood penetration through a range of periods. All of the masks had excellent anti-synthetic blood penetration ability, allowing them to reduce blood contamination of the medical staff during treatment. With a prolonged breathing simulation, masks continued to resist this substitute, demonstrating that the respiratory process did not affect it. 

**Figure 5:**
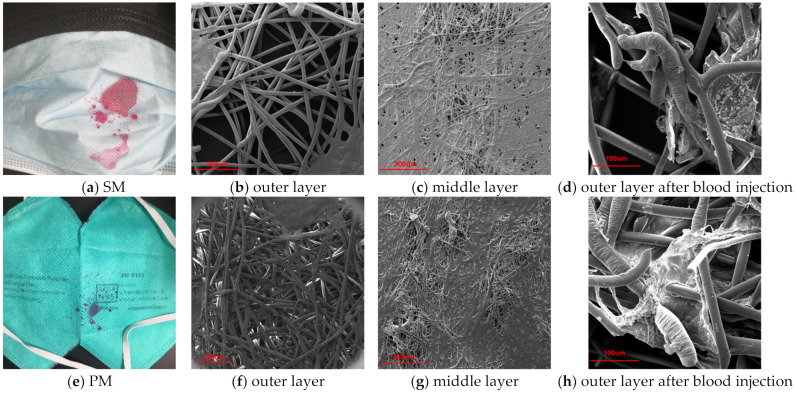
Surface morphology of the mask after blood injection.

The surface morphology of masks before and after a blood spray is displayed in Figure 5. As the spots dried, pores in the collagen fibre network were firmly filled, encasing the fibres in blood-borne material. Due to the superior chemical resistance of the polypropylene material, no noticeable alterations were found in the fibres [38]. Blood was seen to enter the outer layer of the medical surgical mask when it was ejected, accumulating in the gap between the outer and middle layers due to the structure of the SM. The outermost layer of the spunbond fabric had bigger holes, allowing blood to travel through. While the middle meltblown fabric had fine fibres and minute pores, it was unable to make it as well. Thus, the blood pooled between the two layers of nonwoven fabrics and was mobile. While the PM-type had an outer layer spunbonded with small holes, the surface density was almost twice that of SM, and the layers of the mask were closely laminated by pressure or heat, with nearly no gap; blood was stopped on its surface during the spraying process. Despite the fact that no blood penetrated the inner layer after 8 h of wear, SM posed some risk due to the accumulation of blood within.

### 3.3. Total Bacterial Colonies

**Figure 6:**
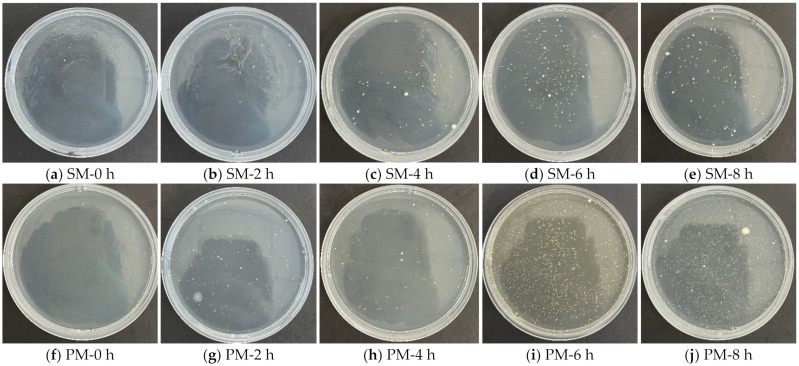
Bacterial colony development after using SM and PM for different lengths of time.

**Figure 7:**
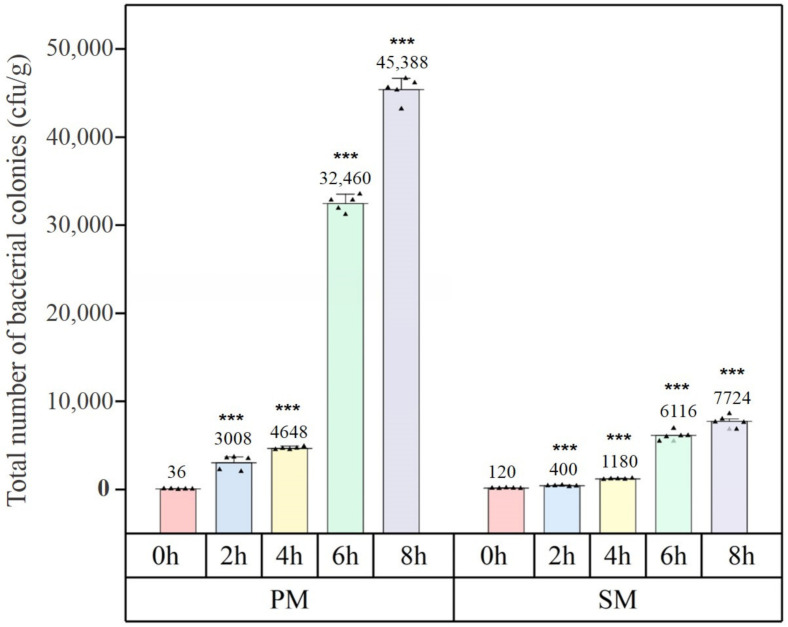
Results of colony formation after mask use. ▲ represents a data point. ***: *p* value < 0.001.

Figure 6 depicts the formation of bacterial colonies for various periods using SM and PM. The number of colonies varied as shown in Figure 7. A tiny number of colonies were identified on both unused masks, but they did not exceed the product limit (200 CFU/g or less). Bacterial colonies from PM samples rose very substantially (*p* < 0.001) with continued use, whereas SM experienced a significant increase beginning at 4 h. After varying periods of use, the overall number of bacterial colonies with SM was considerably less than with PM. During the first four hours of use of both masks, bacterial colonies increased steadily before erupting. 

With continuity in respiration, the relative humidity within the mask increased. The temperature inside the mask was comparable to that of the human body. Consistent with the findings of earlier investigations [16,39], bacteria were prokaryotic organisms that, under optimal temperature and humidity conditions, tend to survive and grow. Compared to PM, SM had a relatively poor fit when worn. At the corners of the nose and face, there were openings through which water vapour might get out. PM was quite airtight and fit the face well, and its nonwoven material had a much larger gram weight, around double that of SM. Therefore, its air and moisture permeability were poor, and it was difficult for vapour to escape the mask. Therefore, inside the PM was more favourable to microbial development, and the number of bacterial colonies was significantly higher than that for SM. If conditions allow, PM should be replaced after two hours, whereas SM should be after four hours.

### 3.4. Breathing Resistance

**Figure 8:**
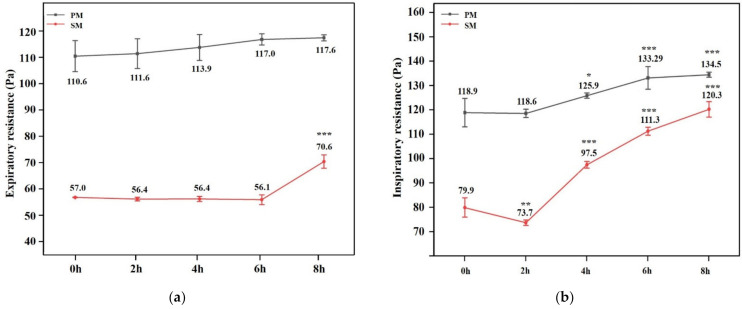
Results of experiments on expiratory and inspiratory resistance. (**a**) expiratory resistance; (**b**) inspiratory resistance. *: *p* value < 0.05, **: *p* value < 0.01, ***: *p* value < 0.001.

Figure 8 illustrates the time-dependent change in respiratory system resistance during exhalation and inhalation. This resistance exhibited an upward trend as the duration of the breathing simulation grew, but did not exceed the standard requirements. In each simulation period, the breathing resistance of PM was greater than that of SM. Only after eight hours of SM usage did the expiratory resistance exhibit a very significant rise compared to unused, but the PM did not substantially alter it with time. After four hours, both types demonstrated a tremendous increase in inhalation resistance, *p* < 0.001 for SM and *p* < 0.05 for PM.

As the duration of mask wear increased, a considerable quantity of moisture gathered inside the mask, slowly creating droplets and making it damp. Some pores were clogged by water vapour, which decreased the air exchange efficiency and gradually raised breathing resistance. The minute ventilation required for strenuous total-body activity was 10 to 15 times higher than typical [40]. When the human body was engaged in intense work, the needed air volume and respiratory rate increased. The prolonged use of a respirator may cause fatigue, increased CO_2_ levels, and decreased O_2_ levels, resulting in hypercapnia or hypoxaemia, which can lead to headaches [41]. Likewise, hypoxaemia was observed with N95 masks, especially with greater respiratory rate and increased incidence of discomfort in the chest during a four-hour use [42], which was similar to our findings. High-resistance breathing resulted in respiratory exhaustion in muscles and physical pain. When contaminants are present in the external environment, it would be simpler for them to enter the human body through the gap, increasing the risk of leakage. It is not advisable to wear the mask for more than two hours due to inspiratory resistance.

### 3.5. Infrared Imaging of Facial Skin Temperature

**Figure 9:**
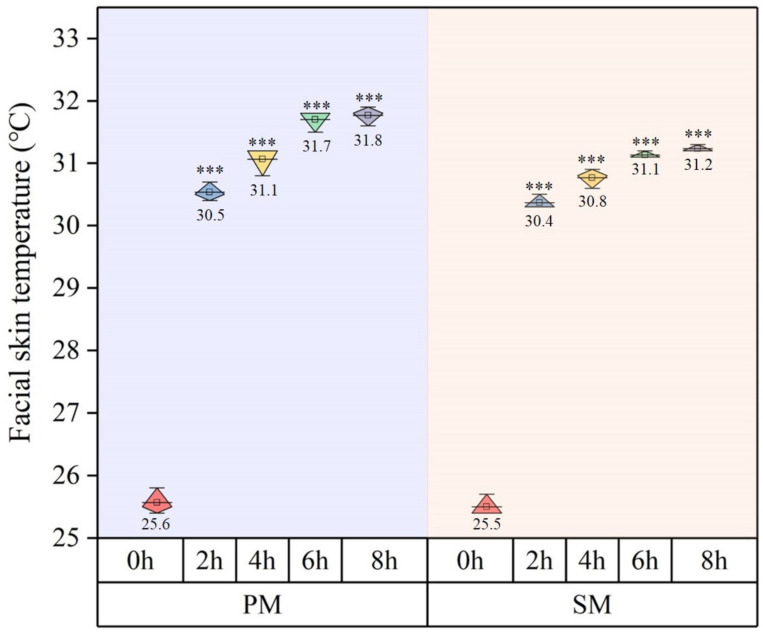
Results of experiments on facial skin temperature after removing masks. ***: *p* value < 0.001.

**Figure 10:**
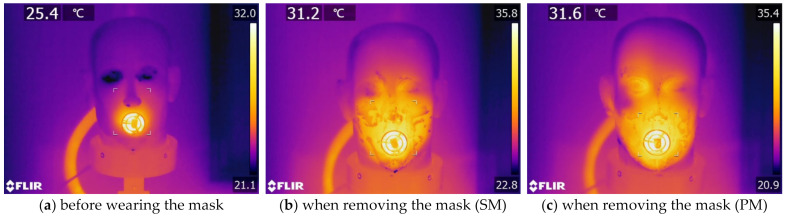
Infrared thermal images of skin temperature after wearing a mask for 8 h (the average temperature of the lips, nose, and cheeks is displayed in degrees Celsius in the upper-left corner).

Figure 9 depicts the facial temperature changes in the head model. As for Figure 10, it displays thermal infrared-derived maps of both face masks, SM- and PM-type, before and after 8 h of use. The face temperature continued to increase after using both, with highly significant differences (*p* < 0.001) between conditions of wearing one or not doing it. There was no substantial change in temperature after six hours. After using one for two hours, the surface temperature of both types increased significantly by 4.9 °C. After eight-hour use, the temperature of SM was 31.2 °C, a rise of 5.7 °C, while that of PM was 31.8 °C, a rise of 6.2 °C.

**Figure 11:**
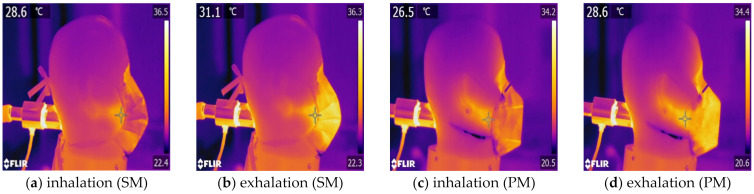
Lateral view, thermal infrared images of breathing while wearing a mask for 8 h (the temperature in degrees Celsius at the marker is indicated in the upper-left corner).

While breathing, thermal infrared images, lateral views, of SM and PM were captured (see Figure 11). From a side perspective view of the thermal infrared imaging, it was fascinating to note that both masks exhibited leakage due to improper fitting. Because of its poor sealing, SM does not conform closely to the face at its sides, and the skin temperature at its edges was substantially greater than that of PM. Additionally, warm water drips could be detected running from the gap under the chin of SM. Healthcare workers can immediately visualise face mask fit modifications using infrared thermography. It solves the issue of long test times and the need for specialty airtight test chambers for healthcare workers in conventional face mask airtightness testing [43].

Due to the particularly good liquid barrier characteristics of the mask, inhaled water vapour at the surface of the head mould cannot exit smoothly, resulting in the formation of a considerable amount of moisture on both the face and mask. The human face contains highly concentrated temperature sensors [44], making it the most sensitive part of the body; hence, an increase there is likely to cause thermal pain [45], skin itching, and other symptoms [46,47,48]. In addition, airway cells that thrive at lower heat might proliferate abnormally in high humidity and temperature [49]. In-mask temperature and humidity could justify the time to wear masks [50]. Given the large rise in temperature, it is suggested the mask be replaced every two hours so that the facial skin can cool down. The maximum wear time should not exceed 4 h.

### 3.6. Moisture Permeability of Mask

**Figure 12:**
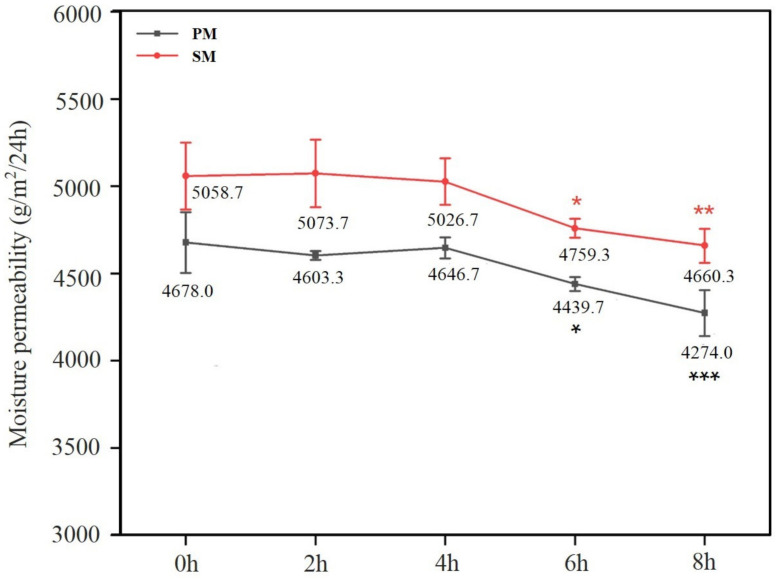
Results of experiments on moisture permeability. *: *p* value < 0.05, **: *p* value < 0.01, ***: *p* value < 0.001.

According to the experimental data, the moisture permeability of SM was better than that of PM. With the extension of time, it showed a downward trend. There was no significant difference in moisture permeability during four hours of mask-wearing. After six hours of breathing simulation, it decreased significantly, as shown in Figure 12. It was lowered by 5.92% for SM, while it was reduced by 5.09% for PM.

Water vapour passes through the fabric in three broad ways [51]: water molecules reach the opposite side via the interior of the fibre; they penetrate to that side with the assistance of wetting between the yarns; and water pressure drives them to move through the pores between the fibres. With the advances in the duration of the respiratory simulation, these molecules entered the mask and adhered to the fibre mesh, which blocked part of the pores. The channel for water molecules through the fabric decreased, as did the moisture permeability. After four hours, the moisture permeability reduced in the mask, and the water vapour could not be discharged in time, which led to an increase in temperature and humidity there. After wearing a mask and walking quickly for one hour, the temperature inside it has gone up from 32 °C to 33.5 °C, and the humidity was about 55% to 90%, respectively [52]. Excessive heat and humidity may cause dyspnea, and water droplets generated when the moisture is high will adhere to the inner layer of the mask or the human face, further increasing the discomfort while wearing one [53]. Because electret technology is adopted in most masks, humidity getting too high would cause dissipation of charge inside, weaken the electrostatic adsorption capacity, and finally lead to a decline in their protective performance. Regarding moisture permeability, it is suggested that masks be replaced after four-hour use.

### 3.7. Optimal Wearing Time for Two Types of Masks

**Figure 13:**
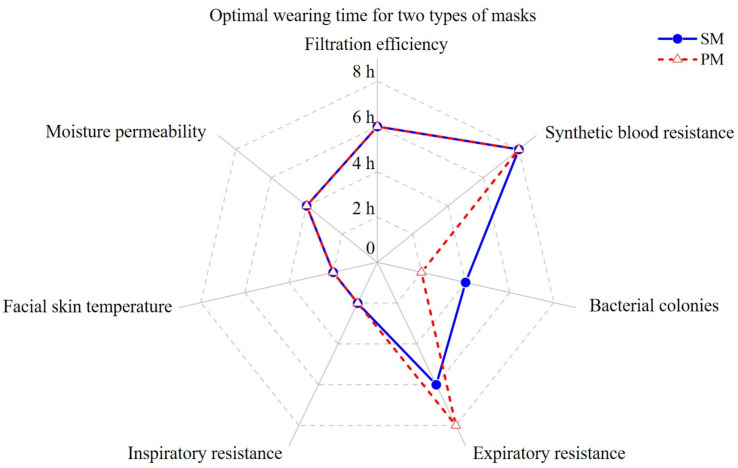
Optimal wearing time for two types of masks.

The optimal wear time for each indicator specific to both types of masks is summarised in Figure 13. In terms of protection, both maintained good filtration of particles and liquid barrier capabilities after six hours of simulated wearing modes, ensuring the safety of the medical staff. However, the number of bacterial colonies using PM and SM grew considerably after two and four hours, respectively. As for comfort, the inspiratory resistance of masks increased significantly after two hours and the moisture permeability decreased after four hours. At the same time, the temperature of the face, mouth, and nose covered by the masks went up substantially after two hours.

## 4. Conclusions

Combining the safety and comfort of masks, it is advised that healthcare workers replace their masks every two hours, not more than four hours if conditions allow. Even though surgical masks are more comfortable than medical respirators, employees are encouraged to wear respirators in COVID-19 risk situations due to their weak protective properties and poor fit to the human face.

This study was conducted in a controlled laboratory environment where the temperature and humidity were kept at 20.0 ± 2.0 °C and 65.0 ± 4.0% to assure constant test conditions for wearing a mask and to minimise errors; thus, it may not fully reflect real-world situations. Extremes in outdoor temperature and humidity, as well as the actual labour intensity faced by medical staff, can significantly affect mask samples and reduce their effective life.

## Figures and Tables

**Table 1:** Basic characteristics of two types of masks.

Number	Sample Name	Product Type	Manufacturer	Material	Code
1	Surgical Face Mask	Surgical mask	Tiger Medical Products Ltd. (Shanghai, China)	Polypropylene	SM
2	Healthcare Particulate Respirator	Medical respirator	3M (St. Paul, MN, USA)	Polypropylene	PM

**Table 2:** Results of synthetic blood penetration resistance after various mask use periods.

Time	SM	PM
0 h	No penetration	No penetration
2 h	No penetration	No penetration
4 h	No penetration	No penetration
6 h	No penetration	No penetration
8 h	No penetration	No penetration

## Data Availability

Data are contained within the article.

## References

[1] Militky J., Novak O., Kremenakova D., Wiener J., Venkataraman M., Zhu G., Yao J., Aneja A. (2021). A review of impact of textile research on protective face masks. Materials.

[2] Anderson R.M., Heesterbeek H., Klinkenberg D., Hollingsworth T.D. (2020). How will country-based mitigation measures influence the course of the COVID-19 epidemic?. Lancet.

[3] Chen J., Wang R., Wang M., Wei G.-W. (2020). Mutations strengthened SARS-CoV-2 infectivity. J. Mol. Biol..

[4] Galloway S.E., Paul P., MacCannell D.R., Johansson M.A., Brooks J.T., MacNeil A., Slayton R.B., Tong S., Silk B.J., Armstrong G.L. (2021). Emergence of SARS-CoV-2 B.1.1.7 lineage—United States, 29 December 2020–12 January 2021. MMWR Morb. Mortal. Wkly. Rep..

[5] Li J.Y., You Z., Wang Q., Zhou Z.J., Qiu Y., Luo R., Ge X.Y. (2020). The epidemic of 2019-novel-coronavirus (2019-NCoV) pneumonia and insights for emerging infectious diseases in the future. Microbes Infect..

[6] Abobaker A., Alzwi A. (2020). The eye: A possible new route of infection in COVID-19. Disaster Med. Public Health Prep..

[7] Nguyen L.H., Drew D.A., Graham M.S., Joshi A.D., Guo C.-G., Ma W., Mehta R.S., Warner E.T., Sikavi D.R., Lo C.-H. (2020). Risk of COVID-19 among front-line health-care workers and the general community: A prospective cohort study. Lancet Public Health.

[8] Liao M., Liu H., Wang X., Hu X., Huang Y., Liu X., Brenan K., Mecha J., Nirmalan M., Lu J.R. (2021). A technical review of face mask wearing in preventing respiratory COVID-19 transmission. Curr. Opin. Colloid Interface Sci..

[9] O’Dowd K., Nair K.M., Forouzandeh P., Mathew S., Grant J., Moran R., Bartlett J., Bird J., Pillai S.C. (2020). Face masks and respirators in the fight against the COVID-19 pandemic: A review of current materials, advances and future perspectives. Materials.

[10] Li X., Ding P., Deng F., Mao Y., Zhou L., Ding C., Wang Y., Luo Y., Zhou Y., MacIntyre C.R. (2022). Wearing time and respiratory volume affect the filtration efficiency of masks against aerosols at different sizes. Environ. Technol. Innov..

[11] Han J., Guan X., Lin J., Wang L., Lin J., Gao X. (2022). Research on efficacy of masks under different simulated wearing modes. Chin. J. Nosocomiology.

[12] (2021). Notice on Printing and Distributing Technical Guidelines for Novel Coronavirus Infection Prevention and Control in Medical Institutions. Bulletin of the National Health Commission of the People’s Republic of China.

[13] Li L., Niu M., Zhu Y. (2020). Assessing the effectiveness of using various face coverings to mitigate the transport of airborne particles produced by coughing indoors. Aerosol Sci. Technol..

[14] Guidelines for the Use of Common Medical Protective Equipment in the Prevention and Control of Novel Coronavirus Pneumonia (Trial Implementation). http://www.nhc.gov.cn/yzygj/s7659/202001/e71c5de925a64eafbe1ce790debab5c6.shtml.

[15] World Health Organization COVID-19: Occupational Health and Safety for Health Workers: Interim Guidance. https://www.who.int/publications/i/item/WHO-2019-nCoV-HCW_advice-2021-1.

[16] Armand Q., Whyte H.E., Verhoeven P., Grattard F., Leclerc L., Curt N., Ragey S.P., Pourchez J. (2022). Impact of Medical Face Mask Wear on Bacterial Filtration Efficiency and Breathability. Environ. Technol. Innov..

[17] Varanges V., Caglar B., Lebaupin Y., Batt T., He W., Wang J., Rossi R.M., Richner G., Delaloye J.-R., Michaud V. (2022). On the Durability of Surgical Masks after Simulated Handling and Wear. Sci. Rep..

[18] Rebmann T., Carrico R., Wang J. (2013). Physiologic and other effects and compliance with long-term respirator use among medical intensive care unit nurses. Am. J. Infect. Control..

[19] Liu C., Li G., He Y., Zhang Z., Ding Y. (2020). Effects of wearing masks on human health and comfort during the COVID-19 pandemic. IOP Conf. Ser. Earth Environ. Sci..

[20] Shechtman L., Ben-Haim G., Ben-Zvi I., Steel L., Ironi A., Huszti E., Chatterji S., Levy L. (2022). Physiological Effects of Wearing N95 Respirator on Medical Staff During Prolong Work Hours in Covid-19 Departments. J. Occup. Environ. Med..

[21] Wangsan K., Sapbamrer R., Sirikul W., Panumasvivat J., Surawattanasakul V., Assavanopakun P. (2022). Effect of N95 Respirator on Oxygen and Carbon Dioxide Physiologic Response: A Systematic Review and Meta-Analysis. Int. J. Environ. Res. Public Health.

[22] Gupta D. (2020). Living with in-mask micro-climate. Med. Hypotheses.

[23] Martel T., Orgill D.P. (2020). Medical device-related pressure injuries during the COVID-19 pandemic. J. Wound Ostomy Cont. Nurs..

[24] Park S., Han J., Yeon Y.M., Kang N.Y., Kim E. (2021). Effect of face mask on skin characteristics changes during the COVID-19 pandemic. Skin Res. Technol..

[25] Gefen A., Ousey K. (2020). Update to device related pressure ulcers: SECURE prevention. COVID-19 face masks and skin damage. J. Wound Care.

[26] Barnawi G.M., Barnawi A.M., Samarkandy S. (2021). The association of the prolonged use of personal protective equipment and face mask during COVID-19 pandemic with various dermatologic disease manifestations: A systematic review. Cureus.

[27] Montero-Vilchez T., Cuenca-Barrales C., Martinez-Lopez A., Molina-Leyva A., Arias-Santiago S. (2021). Skin adverse events related to personal protective equipment: A systematic review and meta-analysis. J. Eur. Acad. Dermatol. Venereol..

[28] Ong J.J.Y., Bharatendu C., Goh Y., Tang J.Z.Y., Sooi K.W.X., Tan Y.L., Tan B.Y.Q., Teoh H.-L., Ong S.T., Allen D.M. (2020). Headaches associated with personal protective equipment—A cross-sectional study among frontline healthcare workers during COVID-19. Headache J. Head Face Pain.

[29] Lee H.P., Wang D.Y. (2011). Objective assessment of increase in breathing resistance of N95 respirators on human subjects. Ann. Occup. Hyg..

[30] Tcharkhtchi A., Abbasnezhad N., Seydani M.Z., Zirak N., Farzaneh S., Shirinbayan M. (2021). An Overview of Filtration Efficiency through the Masks: Mechanisms of the Aerosols Penetration. Bioact. Mater..

[31] Zhu N., Zhang D., Wang W., Li X., Yang B., Song J., Zhao X., Huang B., Shi W., Lu R. (2020). A novel coronavirus from patients with pneumonia in China, 2019. N. Engl. J. Med..

[32] Stadnytskyi V., Bax C.E., Bax A., Anfinrud P. (2020). The airborne lifetime of small speech droplets and their potential importance in SARS-CoV-2 transmission. Proc. Natl Acad. Sci. USA.

[33] Ogbuoji E.A., Zaky A.M., Escobar I.C. (2021). Advanced research and development of face masks and respirators pre and post the coronavirus disease 2019 (COVID-19) pandemic: A critical review. Polymers.

[34] Yovcheva T.A., Mekishev G.A., Marinov A.T. (2004). A percolation theory analysis of surface potential decay related to corona charged polypropylene (PP) electrets. J. Phys. Condens. Matter..

[35] Zhang X., Liu J., Zhang H., Wang Y., Jin X. (2020). Preparation technology and research status of nonwoven filtration materials for individual protective masks. J. Text. Res..

[36] Rengasamy S., Eimer B.C. (2013). N95-companion measurement of cout/cin ratios for two N95 filtering facepiece respirators and one surgical mask. J. Occup. Environ. Hyg..

[37] Seidi F., Deng C., Zhong Y., Liu Y., Huang Y., Li C., Xiao H. (2021). Functionalized masks: Powerful materials against COVID-19 and future pandemics. Small.

[38] Guan X., Han J., Lin J., Wang L., Lin J., Gao X. (2022). Research on anti-blood penetration performance of different types of masks. Chin. J. Infect. Control.

[39] Park A.-M., Khadka S., Sato F., Omura S., Fujita M., Hashiwaki K., Tsunoda I. (2022). Bacterial and fungal isolation from face masks under the COVID-19 pandemic. Sci. Rep..

[40] Johnson B.D., Aaron E.A., Babcock M.A., Dempsey J.A. (1996). Respiratory muscle fatigue during exercise: Implications for performance. Med. Sci. Sports Exerc..

[41] Madan M., Madan K., Mohan A., Hadda V., Tiwari P., Mittal S. (2021). Personal protective equipment and particulate filter use during the COVID-19 pandemic: “acidotic times”. Arch. Bronconeumol..

[42] Lim E., Seet R., Lee K.H., Wilder-Smith E.P.V., Chuah B., Ong B. (2010). Headaches and the N95 face-mask amongst healthcare providers. Acta Neurol. Scand..

[43] Qing Y.E., Xi Y. (2021). Progress in fit testing of respirators. Chin. J. Nosocomiology.

[44] Nakamura M., Yoda T., Crawshaw L.I., Kasuga M., Uchida Y., Tokizawa K., Nagashima K., Kanosue K. (2013). Relative importance of different surface regions for thermal comfort in humans. Eur. J. Appl. Physiol..

[45] Scarano A., Inchingolo F., Lorusso F. (2020). Facial skin temperature and discomfort when wearing protective face masks: Thermal infrared imaging evaluation and hands moving the mask. Int. J. Environ. Res. Public Health.

[46] Foo C.C.I., Goon A.T.J., Leow Y.-H., Goh C.-L. (2006). Adverse skin reactions to personal protective equipment against severe acute respiratory syndrome—A descriptive study in Singapore. Contact Dermat..

[47] Zuo Y., Hua W., Luo Y., Li L. (2020). Skin reactions of N95 masks and medial masks among health-care personnel: A self-report questionnaire survey in China. Contact Dermat..

[48] Szepietowski J.C., Matusiak Ł., Szepietowska M., Krajewski P.K., Bialynicki-Birula R. (2020). Face mask-induced itch: A self-questionnaire study of 2315 responders during the COVID-19 pandemic. Acta Derm. Venereol..

[49] Carpagnano G.E., Lacedonia D., Spanevello A., Cotugno G., Saliani V., Martinelli D., Foschino-Barbaro M.P. (2015). Is the exhaled breath temperature in lung cancer influenced by airways neoangiogenesis or by inflammation?. Med. Oncol..

[50] Cherrie J.W., Wang S., Mueller W., Wendelboe-Nelson C., Loh M. (2019). In-mask temperature and humidity can validate respirator wear-time and indicate lung health status. J. Expo. Sci. Environ. Epidemiol..

[51] Yilma K.T., Limeneh D.Y. (2021). Review on moisture management finish: Mechanism and evaluation. J. Nat. Fibers.

[52] Roberge R.J., Kim J.-H., Benson S. (2012). N95 filtering facepiece respirator deadspace temperature and humidity. J. Occup. Environ. Hyg..

[53] Lee K.-P., Yip J., Kan C.-W., Chiou J.-C., Yung K.-F. (2020). Reusable face masks as alternative for disposable medical masks: Factors that affect their wear-comfort. Int. J. Environ. Res. Public Health.

